# SEC16B identified as a regulator of VLDL secretion and lipid accumulation in the liver

**DOI:** 10.1172/JCI208521

**Published:** 2026-07-15

**Authors:** Hossein Ardehali

**Affiliations:** Department of Medicine and Sarver Heart Center, University of Arizona College of Medicine, Tucson, Arizona, USA.

## Abstract

The liver plays a major role in regulating the metabolic fate of lipids and facilitates lipid secretion to peripheral organs in the form of VLDLs or lipid storage in lipid droplets (LDs). Hepatic regulation of excess lipids profoundly influences the development of atherosclerosis; thus, uncovering the regulatory mechanisms underlying lipid storage and secretion pathways may reveal additional therapeutic targets. In this issue of the *JCI*, Lu et al. identified a pathway involving SEC16B, showing that this protein functions as a lipid-responsive regulator and mediates VLDL secretion and LD formation to maintain lipid homeostasis. They also demonstrated that a reduction in SEC16B reduced serum lipid levels and atherosclerotic plaque area in *Ldlr^–/–^* mice. These results indicate that SEC16B connects VLDL and LD metabolism, positioning SEC16B as a potential therapeutic avenue for atherosclerosis.

## Role of the liver in lipid homeostasis

**Figure 1:**
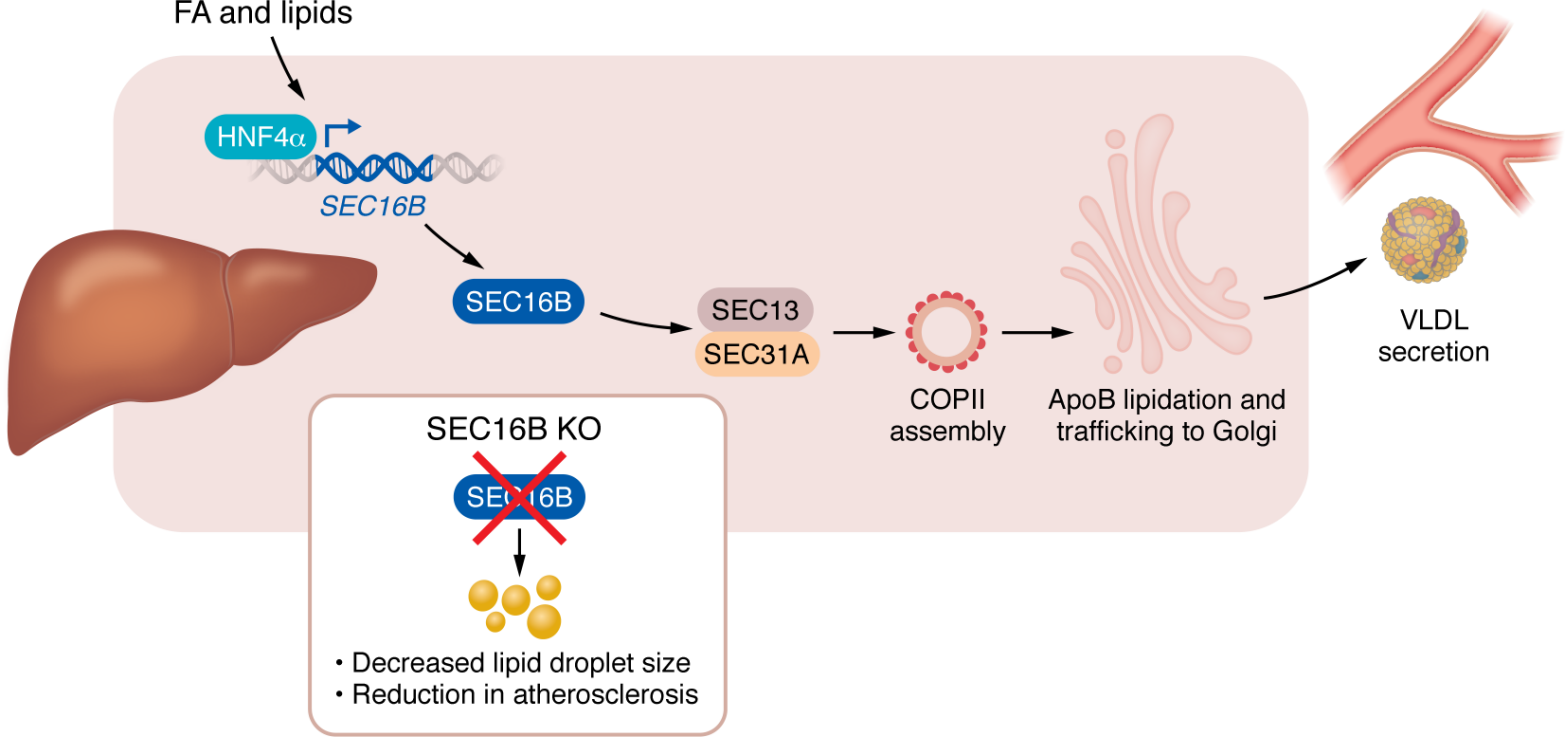
SEC16B maintains hepatic lipid homeostasis by regulating VLDL secretion and LD formation. GWAS identified associations between SEC16B expression and serum cholesterol levels, obesity, and BMI. Here, Lu et al. (5) identified that SEC16B expression increased in response to FA and lipids and was controlled by the transcription factor HNF4α, which regulates expression of key hepatic genes. Using a combination of human hepatocytes and mouse models, they determined that SEC16B KO blocked VLDL secretion from the liver by disrupting the interaction of the COPII components SEC13 and SEC31A. SEC16B KO was also associated with decreased LD size and reduced atherosclerotic plaque formation. Thus, SEC16B represents a link between VLDL secretion and hepatic lipid storage that could potentially be targeted in atherosclerosis and metabolic disorders.

Dysregulation of lipid metabolism gives rise to many chronic disorders, including atherosclerosis and fatty liver disease (1). It is estimated that nearly half of all healthy adults are affected by a degree of atherosclerosis, which can lead to cardiovascular disease (2). The catalyzing event of atherosclerosis is the transportation of lipids, primarily in the form of LDL, to the subendothelial space, where it is engulfed by macrophages, resulting in the formation of foam cells. Although LDL is a key player in the development of atherosclerosis, there is also support for the role of elevated VLDLs and other triglyceride-rich lipoproteins in atherosclerosis progression (3). Thus, recent studies have led to the development of drugs that target VLDL metabolism to treat hyperlipidemia; however, these drugs are associated with the adverse effect of lipid accumulation in the liver as lipid droplets (LDs) and eventual development of fatty liver disease (4). In this issue of the *JCI*, Lu et al. (5) present evidence for a link between VLDL metabolism and secretion and suggest that understanding this link may lead to the development of better and safer therapies for atherosclerosis (Figure 1).

Over the past few decades, several studies have investigated lipid regulation and metabolism in different organs, and the liver has emerged as the major organ in lipid secretion and storage (6). The liver regulates lipid homeostasis through de novo lipogenesis, fatty acid (FA) oxidation, lipid storage as LDs, and delivery of lipids to peripheral tissues in the form of VLDLs. Additionally, FAs in the liver are esterified to a glycerol backbone to generate triglycerides (TGs), which are then either metabolized through the FA oxidation in the mitochondria or released through secretion of VLDLs. These TGs are derived from plasma uptake of FAs released from adipose tissue (70%–80%), TG-rich chylomicron remnants (5%–10%), or de novo lipogenesis (5%–30%) (7). When TG synthesis exceeds utilization, LDs are generated in the ER and accumulate in the cytoplasm (8, 9).

## Mechanisms of VLDL and LD biogenesis

VLDL generation begins with the addition of lipids to apolipoprotein B (ApoB) within the ER. This process, known as lipidation, is mediated by microsomal triacylglycerol transfer protein (MTTP). Following additional lipidation steps, newly formed VLDLs then exit the ER and fuse with Golgi bodies, a rate-limiting step in VLDL secretion, for eventual secretion into the bloodstream. While the functions of proteins involved in the synthesis and maturation of VLDLs have been well characterized (10), the mechanism underlying VLDL transport from the ER to the Golgi is not well understood. Recent studies have established that given the large size of VLDL molecules, the canonical coat protein complex II–mediated (COPII-mediated) protein secretory pathway is not involved in VLDL release from the ER (10).

LDs are intracellular organelles most commonly found in the cytoplasm, but also in the nucleus and ER lumen, with versatile functions extending beyond energy storage (11). Generation of LDs begins with lipid accumulation in the ER bilayer until an oil lens is formed and eventually buds to form a nascent LD that can expand by TG incorporation and fusion with other LDs (12). An imbalance between lipid synthesis and VLDL secretion generally leads to formation of LDs, and LD buildup can lead to the development of fatty liver disease and eventually liver damage and cirrhosis (13). Thus, increasing VLDL secretion should lead to lower LD formation and hepatic liver accumulation; however, the majority of people with dyslipidemia have elevated serum VLDLs and fatty liver, suggesting that the liver has limited capacity to secrete VLDLs (14). There are reports of close functional interaction between LDs and VLDLs (15, 16); however, the mechanisms of these interactions are not known.

## SEC16B mediates the interaction between VLDLs and LDs

Lu and colleagues identified SEC16B as a regulator of both VLDL and LD metabolism (5). Previous GWAS identified an association between the minor allele of a SNP (rs6682862, chr1:177969302) located in the promoter region of *SEC16B* and serum cholesterol levels (17, 18) and determined that *SEC16B* SNPs are strongly associated with obesity and BMI (19). Using this information, the authors established that this minor *SEC16B* allele is correlated with increased hepatic expression of *SEC16B*. In vitro and in vivo assays revealed that HNF4A is a key regulator in *SEC16B* expression and showed that SEC16B was upregulated in response to FAs and lipids. Lu and colleagues generated whole-body and liver-specific *Sec16b*-KO mouse models, both of which exhibited lower serum TG levels. Additionally, the liver-specific *Sec16b-*KO model showed lipid accumulation that was not observed in whole-body *Sec16b*-KO mice.

Based on these findings, Lu et al. hypothesized that loss of *Sec16b* may block VLDL secretion from the liver. The authors discovered that SEC16B regulates ApoB secretion, which appears to be partially independent from MTTP-mediated initial lipidation. Subsequent studies indicated that SEC16B regulated VLDL production by mediating both ApoB lipidation during the generation of VLDL and trafficking nascent VLDL to the Golgi in liver cells. In studies to elucidate the molecular mechanisms by which SEC16B regulates VLDL secretion, the authors showed that knockdown of *SEC16B* in human hepatocytes impaired the interaction between the COPII components SEC13 and SEC31A. This disruption of COPII assembly altered ApoB secretion, which indicates that SEC16B is critical for COPII-mediated VLDL trafficking. Because impaired VLDL formation leads to LD generation, the authors then assessed LD formation and showed that SEC16B was necessary to regulate hepatic LD size in human hepatocytes; however, *SEC16B* deficiency led to smaller LD size. Given that *SEC16B* deficiency impaired VLDL secretion and is expected to produce larger LDs, the smaller LDs observed in liver-specific *SEC16B-*KO hepatocytes are likely the result of a mechanism independent of impaired VLDL secretion. Through a series of sophisticated studies in control and in liver-specific *SEC16B-*KO hepatocytes, the authors showed that SEC16B partially localized to ER-LD contact sites and regulated LD expansion by modulating the translocation of LD-associated ER proteins (Figure 1). Finally, they provide clinical implications for their findings in *Ldlr^–/–^* models of atherosclerosis, showing that: (a) constitutive *Sec16b* deficiency reduced atherosclerosis without causing lipotoxicity, (b) acute deletion of hepatic *Sec16b* slowed down the progression of atherosclerosis without significant effects on the liver, and (c) SEC16B is not a key player in the development of metabolic dysfunction–associated steatohepatitis because hepatic *Sec16b* deletion did not alter the progression of this disorder in mouse models.

These studies are innovative and will advance the field considerably. Lipid storage can be detrimental to the liver and lead to fatty liver disease, so regulation of lipid secretion in the form of TG-rich VLDLs to supply the peripheral tissue is an important, yet understudied, physiological process. The findings by Lu et al. provide a mechanism by which these two processes communicate with each other. They demonstrated that SEC16B regulates hepatic lipid metabolism that increases both VLDL secretion and the expansion of LDs. By doing so, SEC16B regulates lipid flux and maintains lipid homeostasis in the liver and in the bloodstream. Most importantly, deletion of SEC16B in preclinical models led to a reduction in serum lipid levels and decreased the likelihood of developing an atherosclerotic plaque. Given that atherosclerotic vascular disorders are now the most common cause of death in the world, targeting SEC16B may become an important therapeutic approach to reduce the incidence of such a common disease.

These studies complement another recent publication on the role of SEC16B in hepatic lipid homeostasis (20). Wang et al. showed that SEC16B plays a critical role in ApoB-containing lipoproteins from the liver and is an important regulator of COPII machinery. Through a series of bioinformatic, genome-wide correlation, tissue-specific expression, and single-cell analyses, they identified SEC16B as a selective regulator of lipid transport and lipoprotein generation. They then employed functional studies and prediction analysis using AI to show that SEC16B puts a brake on the COPII-mediated role in lipoprotein export. Mining clinical databases, they then showed that SEC16B is linked to metabolic diseases, and its expression is regulated by HNF4A. Finally, they showed that SEC16B deletion in mice protects against atherosclerosis formation and decreases serum levels of ApoB, TG, and cholesterol. These data support Lu et al.’s findings and indicate the SEC16B is a regulator of lipoprotein (VLDL) export and may be a potential target for therapy in atherosclerosis and metabolic disorders.

## Unanswered questions

Although these studies are interesting and provide a new pathway for the regulation of lipid secretion and storage, there are some questions that remain to be answered. First, in mice lacking *Sec16b*, ApoB was delipidated but still accumulated in the liver ER, which contradicts previous findings that delipidated ApoB gets degraded (21). Further study is needed to determine whether partial or complete lipidation of ApoB has differential effects on its degradation as well as the exact mechanism by which SEC16B plays a role in ApoB lipidation. Second, the mechanism by which SEC16B regulates lipidation of VLDL, whether this process is mediated by LDs, and how it is carried out were not studied in this paper and need to be determined. Third, the mechanism by which mice with liver-specific deletion of *Sec16b* evaded the development of metabolic dysfunction–associated steatotic liver disease remains elusive. It will be interesting to study whether this occurred because SEC16B deficiency impaired LD expansion and led to microvesicular steatosis or if this is due to other processes. Fourth, the functional relationship between SEC16A and SEC16B needs to be better clarified. Both proteins are involved in COPII vesicle assembly and the organization of ER exit sites (22, 23). Finally, it is important to identify the potential side effects of pharmacological inhibition of SEC16B, as such therapy is expected to have anti-atherosclerotic effects.

## Conflict of interest

HA serves as an expert witness on topics related to healthcare.

## Funding support

This work is the result of NIH funding, in whole or in part, and is subject to the NIH Public Access Policy. Through acceptance of this federal funding, the NIH has been given a right to make the work publicly available in PubMed Central.

- NIH grants R01HL180711 and 1R01HL175366.
- Leducq Foundation grant.

## Version 1 07/15/2026

Electronic publication
